# Knowledge, perceptions, and experiences of e-cigarettes among young adults in Cape Town, South Africa: Insights from focus groups to inform regulations and prevention strategies

**DOI:** 10.18332/tpc/190616

**Published:** 2024-09-04

**Authors:** Pakhani Mhazo, Alison Swartz, Taghrid Asfar, Melissa Wallace

**Affiliations:** 1 Division of Social and Behavioral Sciences, School of Public Health and Family Medicine, University of Cape Town, Cape Town, South Africa; 2 Institute of Medical and Biomedical Education, St George's University of London, London, United Kingdom; 3 Department of Public Health Sciences, Miller School of Medicine, University of Miami, Miami, Florida, United States; 4 Sylvester Comprehensive Cancer Center, Miller School of Medicine, University of Miami, Miami, Florida, United States; 5 Cancer Association of South Africa, Cape Town, South Africa

**Keywords:** electronic cigarettes, smoking cessation, qualitative analysis, focus group discussions, South Africa

## Abstract

**INTRODUCTION:**

E-cigarettes have rapidly gained a market share in South Africa and globally. Concerns have been raised over the growing popularity of e-cigarettes among young people, who are frequently drawn to these novel products and are especially targeted by marketers. Using a qualitative method, this study aimed to gain insight into young adults' knowledge, experiences, and perceptions of e-cigarette use in Cape Town, South Africa.

**METHODS:**

We conducted five focus groups (FGs) among students of the University of Cape Town (n=48; 46% females; 54% males; aged 18–25 years). These FGs, which included both e-cigarette users and non-users, were audio-taped, transcribed verbatim, and analyzed thematically using Nvivo 12 software.

**RESULTS:**

Despite their lack of information about the chemical constituents of e-cigarettes and their harm, participants perceived them as healthier than combustible cigarettes. Participants equated the pleasant smell and environmental friendliness of e-cigarettes with safety. The absence of e-cigarette regulation was interpreted as evidence of their safety. Participants indicated that the lack of anti-e-cigarette indoor policies, the deceptive marketing regarding their safety, and their low price compared to combustible cigarettes, had key roles in increasing young people's use of e-cigarettes.

**CONCLUSIONS:**

Findings highlight factors at multiple levels contributing to e-cigarette use among young people in South Africa. Comprehensive strategies for e-cigarette regulation and prevention are needed. Potential strategies include increasing knowledge of e-cigarette harms through evidence-based communication campaigns and strengthening e-cigarette regulations by limiting e-cigarette advertisements, banning vaping in public places, and reducing the flavors used in e-cigarettes.

## INTRODUCTION

The use of e-cigarettes has reached epidemic levels worldwide^1^, particularly among young people. While expected to be less harmful than cigarettes, e-cigarettes emit toxic substances, including nicotine, that irreversibly affect youth’s developing brain, leading to dependence and increased risk of cigarette smoking initiation^2^. E-cigarettes have been promoted as a healthier alternative to combustible cigarette smoking and as a tool for smoking cessation^3,4^. Unlike older adults who consider e-cigarettes primarily as a smoking cessation mechanism, young people are often attracted to e-cigarette appeals and ‘trendiness’^5^. In youth and young adults, in particular, nicotine can harm brain development, which continues until about the age of 25 years (CDC 2020). Evidence suggests that ENDS use among young people is driven in part by misperceptions of its safety. Concern has been raised that e-cigarettes may function as a gateway through which new nicotine users and/or tobacco smokers can be recruited^6-8^, thereby undermining efforts to reduce tobacco consumption.

In South Africa, only a few studies have documented the use of e-cigarettes. In the most recent study, the prevalence of ever e-cigarette use among young adults aged 18–24 years was 7.9% in 2021^9^. E-cigarettes in South Africa are currently classified as medicinal products (smoking cessation aids)^10,11^ and the legislation restricts the sale of nicotine delivery e-cigarettes to pharmacies^12^. However, as indicated by their availability in tobacco stores and specialist e-cigarette distributors across the country, e-cigarettes remain mostly unregulated^13^. The South African e-cigarette market is projected to grow annually by about 2.6% between 2024 and 2029^14^. In addition, the revised *Control of Tobacco Products and Electronic Delivery Systems Bill* which aims to classify and regulate e-cigarettes as tobacco products in South Africa^15^ has not yet been passed. More importantly, e-cigarette marketing strategies and advertisements often target young people^16,17^. University students are frequently attracted to new products and have historically been at the forefront of societal changes in substance use that eventually manifest in the general population^18^. A study in South Africa revealed that nearly 50% of e-cigarette shops or vendors are clustered within a 5 km radius of higher education institutions^19^. It is therefore important to understand student’s knowledge, perceptions, and experiences of e-cigarette use in the South African context.

Most available data on young adults’ perceptions and experiences of e-cigarette usage have been conducted in Europe, Canada, and the United States, and little is known about how young people perceive and engage with e-cigarettes in the South African context. The main objective of the current study is to explore and gain insight into the perspectives and experiences of young adults to guide future efforts to prevent e-cigarette use among young adults. Results from the current study will inform future efforts to regulate e-cigarettes in South Africa.

## METHODS

### Design

A qualitative phenomenological approach was used in this study which was conducted among young adult students (aged 18–25 years) at the University of Cape Town (UCT) campuses. Five mixed-gender focus group discussions (FGs) were conducted between November and December of 2018. The sample comprised e-cigarette users (n=20; defined as any persons who used e-cigarettes for 30 days or more, including former and current vapers, regardless of their use of other tobacco products) and non-users (n=28; individuals who had never used e-cigarettes before, regardless of their use of other tobacco products). This study was approved by the UCT Human Research Ethics Committee (HREC) and the School of Public Health and Family Medicine.

### Participants and recruitment

Purposive sampling was used to recruit participants in this study. Purposive sampling helps to identify and select participants who are information-reach and appropriate to the study^20^. Participants aged 18–25 years (n=48) were recruited through face-to-face interactions at the UCT campuses. The researchers also used referrals from networks of UCT students to recruit more e-cigarette users and non-users. Prospective participants were screened for eligibility through face-to-face interaction, and those recruited via referrals were screened by phone. Eligible participants were scheduled for focus group sessions and provided the time and place for their focus group session. Participants received a 100 ZAR (100 South African Rand about US$5.5) incentive for participating in the study.

### Procedures

Five FGs were conducted in a private conference room at the UCT upper campus, two with e-cigarette users, two with non-users, and one with both users and non-users. Each FG consisted of 8 to 11 participants. FGs allowed for the exploration of participants’ common and divergent viewpoints and experiences. A semi-structured focus group guide developed by the research team explored the following topics: 1) knowledge of e-cigarettes, 2) harm perceptions of e-cigarettes, and 3) general attitudes and experiences of e-cigarette use. Focus groups were moderated by two public health researchers trained in qualitative methods. Each session began with a general discussion on the nature, confidentiality, and general interaction preferences for the group discussion. After explaining the study and obtaining written informed consent, participants completed a brief baseline assessment survey, followed by a focus group discussion. While the FG guides featured particular discussion questions, they retained flexibility in terms of question wording and sequencing, allowing for detailed probing. Each FG lasted for approximately 60 minutes and was audio recorded and afterward transcribed by an independent transcription service. Interview transcripts were distributed to participants for verification of accuracy and consistency with their perspectives.

### Data analysis

Data were analyzed using thematic analysis. Inductive coding was used in the NVivo 12 program to allow themes to emerge from the data. The first author (PM) began by reading the transcripts attentively and creating a codebook. The codebook and transcripts were then forwarded to the second and third authors (AS and MW) to ascertain whether they concurred or disagreed with the generated codes. The initial codes reflected the participants’ most commonly mentioned concepts. Themes were then created by grouping related codes.

## RESULTS

### Participant characteristics

Table 1 summarizes the participant characteristics. Participants were full-time registered students at UCT aged18–25 years (n=48). Overall, 42% of participants used e-cigarettes, while 58% were non-users. Among these, 35% were exclusive e-cigarette users, and 65% were dual users of both e-cigarettes and combustible cigarettes/or other tobacco products. Among e-cigarette users, 65% were male and 35% were female.

**Table 1:** Participant characteristics of a qualitative study on young adults’ knowledge, perceptions and experiences of e-cigarettes in Cape Town, 2018 (N=48)

*Characteristics*	*n*	*%*
**Age** (years)		
18–19	15	31
20–21	12	25
22–23	13	27
24–25	8	17
**Gender**		
Female (users)	22 (7)	46
Male (users)	26 (13)	54
**Race**		
Black	13	27
White	15	31
Mixed race	18	38
Other	2	4
**E-cigarette use status**		
Use (dual-use)	20 (13)	42
Never use	28	58

### Knowledge of e-cigarettes

There were notable differences in knowledge between e-cigarette users and non-users. Unlike users who knew what e-cigarettes are, some non-users admitted that they did not know the name or purpose of these products before they participated in this study. No gender differences in knowledge were found.

*Knowledge sources*

Many participants mainly relied on information from social media, e-cigarette marketers, and social networks, as evidenced by the excerpts below:

*‘Friends and relatives. Like if you have friends and relatives who use e-cigarettes, they have more knowledge, and I just ask them.’* (Female, 23 years, non-user)

*‘I think mostly through people at school. Like other students have them, and they can share what they know.’* (Male, 20 years, e-cigarette user)

‘m*y main sources would be like Instagram. Like I read a lot from the Twisp page and others.’* (Female, 19 years, e-cigarette user)

Despite showing limited knowledge of e-cigarettes, many participants reported that they have never consulted any scientific research or health professionals to learn about these devices. Those who have consulted scientific studies indicated that they accessed these articles from platforms that promote the selling and use of e-cigarettes, as reflected below:

*‘I’m also part of a vape forum called ECIGSSA and they put up a lot of latest research and stuff on there. So yeah, I just sort of look for the latest articles and verify my sources … I’m not just reading someone’s opinion … it’s scientifically backed up.’* (Male, 19 years, e-cigarette user)

*Chemical constituents of e-cigarettes*

Many participants had limited knowledge about the chemical constituents of e-cigarettes. While some participants pointed out nicotine and flavors as the only constituents that they knew, others indicated that such information is not always available to them. Participants indicated that some e-liquid containers do not have information detailing their contents:

*‘They don’t come with ingredients written on them, but who cares about ingredients. I just know that they are cool, and the flavors kinda tell you that they are healthier.’* (Male, 21 years, e-cigarette user)

Some participants admitted that they did not know the constituents of e-cigarette liquid and even asked investigators to tell them instead, as reflected below:

*‘I would like to know the constituents of that. Do you know them? Please tell us now. I want to know their components.’* (Female, 22 years, e-cigarette user)

The above results indicate that most participants had limited knowledge about the chemical constituents of e-cigarettes, and their main sources of information were social media, e-cigarette marketers, and social networks.

### Perceptions of e-cigarettes

Despite having limited knowledge about e-cigarettes and their chemical constituents, most participants perceived e-cigarettes positively. Participants mainly perceived e-cigarettes as ‘healthy’ and ‘classy.’

*E-cigarettes as a ‘healthier’ option than combustible cigarettes*

Many participants in this study (both users and non-users) believed that e-cigarettes are healthier than combustible cigarettes, as is evidenced by the excerpts below:

*‘The water vapor that gets into your lungs can cause problems. But I still think tobacco causes a lot more damage than water vapor that comes with e-cigarettes.’* (Male, 19 years, e-cigarette user)

*‘I know they are slightly bad for you, but they’re still roughly 95% healthier than real cigarettes. I know that they are not 100% good for me, but I know that everyone also eats sugar, and sugar is also terrible for you. So, I decided it’s not that bad.’* (Male, 20 years, e-cigarette user)

Some non-users cited the pleasant aroma of e-cigarettes as an indication that they are healthier than combustible cigarettes:

*‘Just me bumping into a person who is smoking a cigarette; I feel like smelling death when I get that smell. But with e-cigarettes, you think like, “Oh, it’s so sweet”. I feel like the smell is not disgusting like tobacco; it’s nice, and it must be healthy. You can just feel it. They are also clean. Like you don’t drop the stub and leave the environment dirty.’* (Female, 20 years, non-user)

Some participants perceived the lack of e-cigarette regulation in South Africa as evidence that they are a safe alternative to combustible cigarettes:

*‘When you see something not being controlled like this, it means it’s safe. You hear people say “heh, it’s more dangerous”. Why then is it not controlled like tobacco?’* (Male, 20 years, e-cigarette user)

*E-cigarettes as ‘classy’*

Many participants perceived e-cigarettes as classy and associated their use with high social status, as evidenced by the excerpts below:

*‘They are classy as well. You wouldn’t see someone from the township vaping. I mean, everything about it is just cool.’* (Female, 19 years, e-cigarette user)

*‘It carries some type of class with it. So if you vape or not, it shows whether you’re lower class, middle class or upper class … so there’s a gravitation to a higher social status.’* (Female, 22 years, non-user)

Some participants argued that even if e-cigarettes help to promote smoking cessation, they can only help those of higher social and economic status, as poor people cannot afford e-cigarettes:

*‘So if they are claiming that it helps to quit smoking, it’s only a certain class of people that can afford it. Like middle class or upper class. So it’s not open to every smoker.’* (Female, 24 years, non-user)

*E-cigarettes as socially acceptable*

E-cigarettes in this study were generally perceived as more socially acceptable than combustible cigarettes. This is evident in the participants’ experiences of e-cigarette use, as reflected below:

*‘My mommy does not smoke or vape. If I smoke tobacco cigarettes in the house, she tells me to go outside. But if I use my vape inside the house, she’s like, “Oh, okay. It’s fine, whatever”.’* (Female, 22 years, e-cigarette user)

*‘In my hood, young people don’t smoke in front of elders. It’s disrespectful. But if it’s an e-cig, the elders don’t say anything; they sort of understand that it’s safer.’* (Male, 23 years, e-cigarette user)

The above results show that e-cigarettes are perceived as healthy, classy, and socially acceptable compared to combustible cigarettes.

### Experiences of e-cigarette use

Most of the participants’ experiences of e-cigarette use were linked to their perceptions of these devices. Participants’ experiences, which include motivations for use, accessibility, and use of e-cigarettes, are presented in this section.

*Reasons for e-cigarette use*

Despite perceiving e-cigarettes as healthier, most e-cigarette users identified curiosity as their reason to start using e-cigarettes. Participants were mainly attracted to e-cigarettes because of their appeals, flavors, and ‘vape tricks’:

*‘I just saw people vaping, and they looked cool. It smelled nice, it looked fun, they were doing vape tricks and whatever. So I said to myself, “I need to try that cool thing”.’* (Female, 22 years, e-cigarette user)

*‘I was curious because it was cool that people can blow lots of smoke. Now it’s one of my favorite parts about it - I mean nicotine is not that important. But the blowing smoke was pretty cool.’* (Male, 19 years, e-cigarette user)

Some participants claimed to have started using e-cigarettes to try and quit smoking, but their quitting attempts were unsuccessful. Instead of helping them quit, participants claimed e-cigarettes encouraged them to smoke even more and/or to use both e-cigarettes and tobacco:

*‘I started using e-cigarettes thinking maybe I could smoke less. But that thing makes you smoke way more … now I smoke more, and I vape as well.’*

(Female, 21 years, e-cigarette user)

*E-cigarette use in smoking prohibited zones*

Many participants claimed to use e-cigarettes in areas where cigarette smoking is discouraged and/or prohibited, such as indoors (including classrooms) and in public:

*‘I don’t think people mind if you vape it inside. I see people vape it inside malls and stuff. Even the guys who sell them in malls allow customers to try them inside the mall. Smoke sensors don’t detect the vapor. I sometimes do it (vape) inside buildings that have smoke sensors here (on campus), and nothing happens.’* (Male, 25 years, e-cigarette user)

Some participants who were users also mentioned that their exposure at a very young age to e-cigarette advertisements and marketing was behind their initiation into the habit:

*‘I first tried one in grade 8. A friend of mine had one, and I tried it. then, I finally bought one for myself when I was 15 years old. It’s not like they ask if you are old enough like tobacco where you must be 18 to be able to buy …’* (Male, 18 years, e-cigarette user)

*Accessibility of e-cigarettes*

Results show that many people have easy access to e-cigarettes as these products are available in malls, retail shops, and online platforms. However, due to the higher costs of e-cigarettes, some people opt to buy secondhand e-cigarettes from other individuals or online, for relatively lower prices, as evidenced by the information below:

*‘There are so many places to get them now … but I don’t have a lot of money. They can get very expensive to buy, so I usually try and buy secondhand ones, usually from the same online forum where I get my news and stuff about them - they also have a classified section. So people are selling secondhand stuff. So I’ll either buy from them or I’ll buy from a friend. For example, if someone at varsity is selling, then I’ll buy it from them. And I just sell it if I no longer want it.’* (Male, 19 years, e-cigarette user)

*‘This one is a very expensive brand. I bought it secondhand because I can’t afford a brand new one. It’s more like an upgrade to my previous one because I sold my previous one before I bought this one. It’s cheaper that way.’* (Male, 22 years, e-cigarette user)

Some participants have also indicated that they are making a profit from secondhand e-cigarette business in which they buy used e-cigarettes for lower prices and sell them for slightly higher prices:

*‘I also build my coils and stuff. So, I’ve gotten into the whole technical side of it. And I’ve traded stuff. So, like, I’ll buy stuff for cheap and then sell it for more. If it’s faulty, I’ll fix it. And I also make my own flavors and stuff then sell.’* (Male, 20 years, e-cigarette user)

## DISCUSSION

Our results indicated that most young adults perceive e-cigarettes as healthier than combustible cigarettes despite having limited knowledge of their chemical composition and/or their potential risks. Further, participants cited several general characteristics of e-cigarettes, including a nice smell, environmental friendliness (no risk of dropping cigarette butts or ash), and social acceptability, as evidence that e-cigarettes are healthy. The lack of e-cigarette regulations was viewed as an indication that e-cigarettes are safer than combustible cigarettes. For the majority of e-cigarette users in our study, social networks and exposure to e-cigarette advertisement and marketing were the primary sources of information and one of the main reasons for initiating the habit. Participants also utilized their social media accounts to sell and purchase secondhand and counterfeit e-cigarette products. Findings highlight factors at multiple levels contributing to e-cigarette use among young people in South Africa. Comprehensive strategies for e-cigarette regulation and prevention are needed in South Africa. Potential strategies include increasing knowledge of e-cigarette harms through evidence-based communication campaigns and strengthening e-cigarette regulations by limiting e-cigarette advertisements, banning vaping in public places, and reducing the flavors used in e-cigarettes.

Contrary to the scientific explanation that e-cigarettes are safer than combustible cigarettes because they lack various toxicants and carcinogens that are present in combustible cigarettes^21^, most participants viewed e-cigarettes as healthy because of their pleasant smell and cleanliness. Despite perceiving e-cigarettes as healthy, most participants had never read any research articles on the potential risks of e-cigarettes. Rather, they relied on opinions and recommendations provided by marketers and/or other e-cigarette users on social media and other online platforms. It is worth noting that some of the material discovered by participants on e-cigarette marketing platforms corresponds to findings from previous scientific studies. One such example is the assertion that e-cigarettes are 95% safer than combustible cigarettes, which is consistent with reports from Public Health England^22^. However, marketers tend to focus exclusively on studies that report the beneficial effects of e-cigarettes to promote their products^23^. In this study, young adults’ perceptions of e-cigarettes mirrored the advertising claims and information provided by e-cigarette marketers.

Results also suggest that a lack of legislation or related signage creates an impression that e-cigarettes are safe. The fact that e-cigarettes can be sold to anyone regardless of age, and people can use e-cigarettes in places where smoking is generally prohibited, reinforces the belief that e-cigarettes are safe. Minors’ use of e-cigarettes reflects their ease of access, particularly in a country that lacks clarity on the regulation of e-cigarette distribution, sale, and use. While the long-term risks of e-cigarettes are not yet known^24^, some people interpret the lack of e-cigarette regulation as an indication that e-cigarettes are harmless^25^. Thus, the regulatory environment plays a part in influencing youths’ perceptions and behaviors related to e-cigarette use^25,26^. When e-cigarette use is largely unregulated, some people use e-cigarettes in places and/or circumstances where smoking is prohibited, thereby circumventing smoking regulations^4,26^. Most dual and e-cigarette users in this study exploit loopholes in the current tobacco law and the largely unregulated e-cigarette environment in South Africa. This is contrary to the situation in Finland, where e-cigarettes are regulated as tobacco products, and the use of e-cigarettes in smoking-prohibited zones is not allowed^27^. While dual users in this study smoke combustible cigarettes where permitted, they use e-cigarettes where smoking is prohibited. In this case, e-cigarettes allow smokers more sustained access to nicotine and help to maintain the smoking habit as opposed to stopping smoking.

Unlike older adults who use e-cigarettes primarily as smoking cessation mechanisms, e-cigarette use among younger adults is not always associated with quit intentions^4,28^. Most young adults in this study started using e-cigarettes due to curiosity and/or the desire to be regarded as middle class. The availability of secondhand and counterfeit e-cigarette products at lower prices helps individuals who cannot afford the high cost of new products. Secondhand e-cigarette products provide an entrepreneurial opportunity for young people who are involved in the buying and selling of secondhand products. There is a virtual and actual social network around the purchase of secondhand devices, news about e-cigarettes, information, and risks. Such connections make young people feel part of a community for both current users and prospective users to learn about e-cigarettes. While social media and informal conversations function as sources of information^29^, they also provide an opportunity for people to buy and/or sell e-cigarettes. Secondhand and counterfeit e-cigarette products, however, make it difficult for customers to obtain sufficient or legitimate information regarding these items, as the majority of them do not come with packaging or instruction manuals. The sale of secondhand devices and counterfeit liquids on various platforms may present a significant regulatory and public health challenge.

### Limitations

Since the study findings are based on data collected in 2018, this may limit their direct applicability to the 2024 context due to potential changes in young adults’ perceptions, knowledge, and experiences of e-cigarette use over time. However, the study remains the first and only qualitative study to explore young adults’ knowledge, perceptions, and experiences of e-cigarette use in South Africa. Further research may help to assess changes in young adults’ perceptions and experiences over time. Moreover, the regulatory landscape for e-cigarettes in South Africa has remained in a state of flux, with the Tobacco Products and Electronic Delivery Systems Control Bill still yet to be enacted since its initial proposal in 2018. Thus, the insights gleaned from this study may help to inform the ongoing debates around the proposed e-cigarette regulation in the country.

Furthermore, given the fact that the sample was drawn from a single university, the findings may not necessarily be representative of the perspectives and experiences of all young adults in South Africa. However, the study provides some valuable insights into the nuanced realities around e-cigarette use in South Africa. Further quantitative studies may be conducted to yield more representative findings. Future research may also focus on the regulatory and public health aspects of secondhand and counterfeit e-cigarette marketing and use in South Africa.

## CONCLUSIONS

E-cigarette appeals, poor regulation, and information provided by e-cigarette marketers play a role in shaping young people’s risk perception of these products. E-cigarettes’ lack of regulation and social acceptability may encourage their use in circumstances where smoking is generally prohibited or discouraged. Classifying and regulating e-cigarettes as tobacco-related products could not only help to curb the circumvention of smoking restrictions but may also alter the perception that these products are safe. E-cigarette awareness can be increased, especially through utilizing platforms such as social media, where young people access most of the information.

## Data Availability

The data supporting this research are available from the authors on reasonable request.
